# Longitudinal Variations in the *tprK* Gene of Treponema pallidum in an Amoy Strain-Infected Rabbit Model

**DOI:** 10.1128/spectrum.01067-23

**Published:** 2023-06-22

**Authors:** Dan Liu, Rui Chen, Yun He, Yong-jing Wang, Li-Rong Lin, Li-Li Liu, Tian-Ci Yang, Man-Li Tong

**Affiliations:** a Center of Clinical Laboratory, Zhongshan Hospital, School of Medicine, Xiamen University, Xiamen, China; b Institute of Infectious Disease, School of Medicine, Xiamen University, Xiamen, China; Nevada State Public Health Laboratory

**Keywords:** Amoy strain, syphilis, *Treponema pallidum*, tprK gene, variation

## Abstract

Heterogeneous *tprK* sequences have been hypothesized to be an important factor for persistent infection of Treponema pallidum subsp. *pallidum* (T. pallidum) in humans. Previous research has only explored *tprK* diversity using a rabbit model infected with almost clonal isolates, which is inconsistent with the fact that infected human isolates contain multiple heterogeneous *tprK* sequences. Here, we used the T. pallidum Amoy strain with heterogeneous *tprK* sequences to establish a rabbit infection model and explore longitudinal variations in the *tprK* gene under normal infection, immunosuppression treatment, and benzathine penicillin G (BPG) treatment using next-generation sequencing. The diversity of the *tprK* gene was high in all three groups but was highest in the control group and lowest in the BPG group. Interestingly, the overall diversity of *tprK* in all three groups decreased during infection, exhibiting a “more to less” trend, indicating that survival selection may be an important factor affecting *tprK* variation in the later infection stage. BPG treatment appeared to reduce the diversity of *tprK* but increased the frequency of predominant sequence changes, which might facilitate the escape of T. pallidum from the host immune clearance. Furthermore, the original predominant V region sequence did not disappear with disease progression but retained a relatively high proportion within the population, suggesting a new direction for *tprK*-related vaccine research. This study provides insights into longitudinal variations within the highly heterogeneous *tprK* gene sequences of T. pallidum and will contribute to further exploration of the pathogenesis of syphilis.

**IMPORTANCE** The *tprK* variations are an important factor in persistent T. pallidum infection. A nearly clonal isolate has been used previously to investigate the mechanism of *tprK* gene variations; however, clinical T. pallidum isolates in infected humans exhibit multiple heterogeneous *tprK* sequences. Here, we use next-generation sequencing to explore longitudinal variations in the *tprK* gene under normal infection and immunosuppression and benzathine penicillin G treatment in a rabbit model infected with the Amoy strain with heterogeneous *tprK* sequences. The overall diversity of *tprK* in all three groups was high and decreased during infection, exhibiting a “more to less” trend. Benzathine penicillin G treatment reduced the diversity of *tprK* but increased the frequency of predominant sequence changes. Moreover, the original predominant V region sequence did not disappear as the disease progressed but remained at a relatively high proportion within the population. The research results give us a new understanding about *tprK* variation.

## INTRODUCTION

Syphilis, caused by the spirochete Treponema pallidum subsp. *pallidum* (T. pallidum), is a chronic sexually transmitted disease that can persist as a lifelong infection without treatment (1–3). The underlying mechanism of chronic and persistent T. pallidum infection was unknown for a long time (4) until the first whole-genome sequencing of the T. pallidum Nichols strain (~1.13 Mb) provided a new research direction for understanding the pathogenesis of syphilis (5). Following the genome sequencing of many other T. pallidum strains (6–8), including the T. pallidum Amoy strain isolated in our laboratory (9), comparative genomics analysis has revealed that T. pallidum strains exhibit high similarity in their genome sequences and are genetically distinct in limited regions, with the antigen-encoding Treponema pallidum repeat K (*tprK*) gene showing particularly high diversity (10, 11). T. pallidum possesses only a single copy of the *tprK* gene but exhibits heterogenous *tprK* sequences at the intra- and interstrain level (12). Heterogeneity in the *tprK* gene is localized within seven discrete variable (V) regions (V1 to V7), flanked by conserved domains (10, 11). Previous studies have demonstrated that the antibody against TprK facilitates the phagocytosis of T. pallidum (13) and that TprK undergoes antigenic variation in V regions, with minimal or no antibodies detectable against the mutated V sequences, which aids the immune evasion of T. pallidum (14, 15). Therefore, insights into the characterization of *tprK* variations can help us explore the pathogenic mechanism of syphilis.

As early as 1999, Centurion Lara first used rabbit models infected with the Nichols standard strain to investigate variations in the *tprK* gene (16). Subsequently, other studies found that the Nichols strain had only one identical *tprK* gene sequence that varied minimally during infection, which is in substantial contrast to the *tprK* gene in other clinical isolates (12). Although the reason for the reduced variability in Nichols *tprK* is still unclear, this strain was not appropriate for exploring the mechanism of *tprK* gene variation. Subsequently, the clonal isolate Chicago C, derived from Chicago, which maintains high variability during infection, was used to investigate the characterization of *tprK* variations (14, 17, 18). These studies proved that *tprK* variations facilitate the escape of T. pallidum from the host immune clearance and that immune pressure may enhance the accumulation of this variation (14, 18). In addition, the generation of V region diversity is driven by nonreciprocal segmental gene conversion (17). This finding has shed important light on the *tprK* gene.

However, research based on a clonal isolate has not been widely conducted because of the complexity of clone preparation and generation of new mutation sequences in *tprK* during subpassage. More importantly, T. pallidum involved in the natural syphilis infection in humans exhibits heterogeneous *tprK* sequences (3, 19). Thus, further exploration of *tprK* longitudinal variations in strains possessing multiple *tprK* sequences is required to elucidate host-pathogen interactions. Furthermore, technological advances have enabled the use of next-generation sequencing (NGS) to directly analyze the profiles of V sequences in the *tprK* gene from patients with syphilis (2, 3), which can provide more molecular data for the systematic analysis of *tprK* variations.

**FIG 1:**
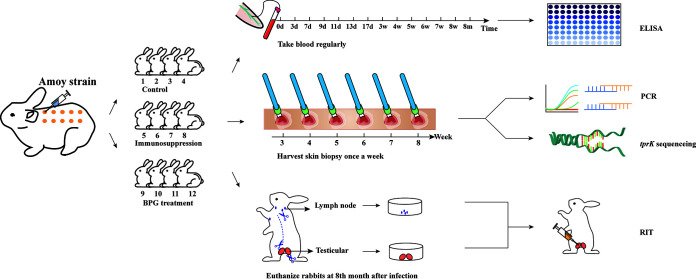
Experimental overview. The following three experimental groups were used in this study: control group, BPG treatment group, and immunosuppression group. BPG, benzathine penicillin G; d, day; w, week; m, month; ELISA, enzyme linked immunosorbent assay; RIT, rabbit infectivity testing.

Therefore, in this study, we use the Amoy strain, which contains multiple *tprK* sequences, to establish a rabbit infection model and analyze variations in the *tprK* gene under normal infection and different interventions (immunosuppression and benzathine penicillin G [BPG] treatment) (Fig. 1). We comprehensively explore the profiles of *tprK* diversity, thereby providing a molecular foundation for the role of the *tprK* gene in immune escape and vaccine research against syphilis.

## RESULTS

### Diversity of *tprK* during Amoy strain infection in the three rabbit infection models.

Based on quality control filtering, 68 samples from the three groups were enrolled in this study (see Table S2 in the supplemental material). Moreover, 561 variants of the seven V regions were extracted from the *tprK* gene, with the lowest and highest number of unique sequences presented in V4 and V6, respectively (Table 1). The control group had the most variants, with up to 412 (ranging from 11 to 191), followed by the immunosuppression group with 315 variants (ranging from 6 to 150), whereas the variability of *tprK* in the BPG treatment group was relatively low, with only 242 variants from seven V regions (ranging from 9 to 101) (Table 1).

**TABLE 1:** Comparative analysis of the variant number of *tprK* genes among three rabbit infection models^*a*^

Peptide	Results by group						Total no. of distinct variants
	Control group		Immunosuppression group		BPG treatment group		
	No. of distinct variants	Ratio (%)	No. of distinct variants	Ratio (%)	No. of distinct variants	Ratio (%)	
V1	16	100.0	10	62.5	12	75.0	16
V2	22	84.6	16	61.5	18	69.2	26
V3	50	67.6	47	63.5	32	43.2	74
V4	11	91.7	6	50.0	9	75.0	12
V5	59	77.6	43	56.6	34	44.7	76
V6	191	67.7	150	53.2	101	35.8	282
V7	63	84.0	43	57.3	36	48.0	75
Total	412	73.4	315	56.1	242	43.1	561

a Only *tprK* variants from skin lesions at third to eighth week after Amoy strain infection are included in this table. The “Total no. of distinct variants” referred to the number of different types of variants in each V region; the same type of variants in different groups are only counted once. Ratio was determined as the “No. of distinct variants in each group” divided by “Total no. of distinct variants.”

**FIG 2:**
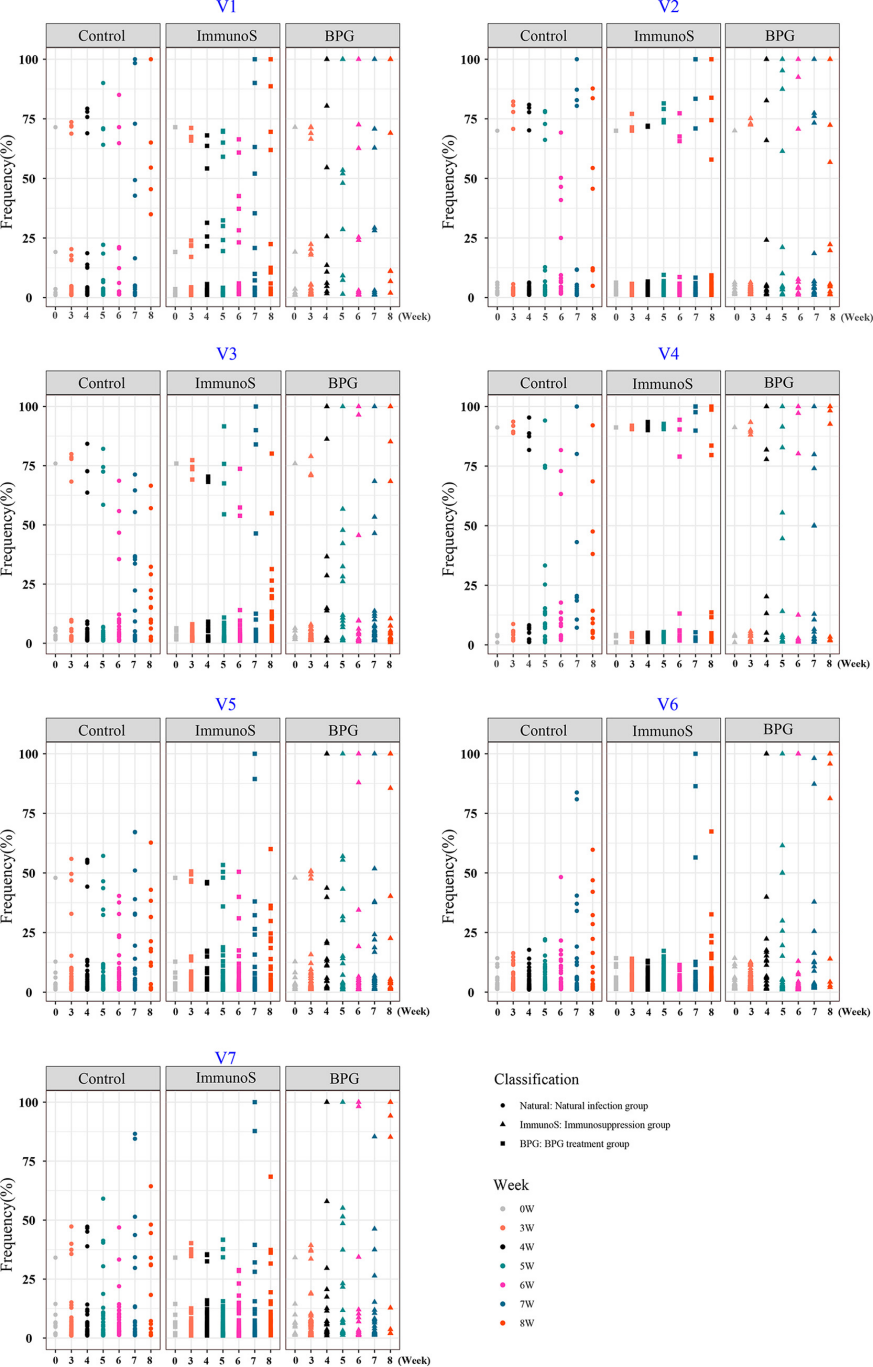
Distribution of distinct sequences in a single V region of *tprK* within each sample in the Amoy inoculum and three rabbit infection models. Week “0” represents the “Amoy inoculum.” BPG, benzathine penicillin G; W, week. Each point represents a variant. The relative frequency of every variant sequence within each variable region in every group was presented with the same color dot. The total frequency of variants in each V regions of a single sample is 100%, and when sequencing results are obtained for a group of four rabbits, the total frequency is 400%.

An analysis of the distribution of distinct sequences in a single V region within each sample showed that the frequency distribution of the different variants in all seven V regions from the Amoy inoculum (week 0) was relatively scattered, and the frequency of predominant sequences (the highest proportion variants in a single sample) was low, especially that of the V6 predominant sequence, which was only 14.2% (Fig. 2). The variation of the *tprK* gene in the control group became high from the fifth week after infection, and the high-frequency predominant sequences were mostly within the range of 20 to 75%, rarely reaching 100%. Conversely, the frequency distribution of different variants in the immunosuppression group changed slightly over the six betamethasone injections. The frequency distribution of the *tprK* gene was almost the same as that of the inoculum (week 0), especially for the predominant sequences in V6 and V7, which remained consistently at approximately 14% and 38%, respectively. However, the frequency of different variants changed greatly after the seventh week (2 weeks after the last betamethasone injection). A few high-frequency predominant sequences appeared, with some reaching 100%. The variation of the *tprK* gene in the BPG treatment group varied significantly during the infection period, that is, the frequency of V predominant sequences increased rapidly, with some reaching 100% from the fourth week after the first penicillin administration (Fig. 2). As the frequency of predominant sequences increased, that of the minor sequences decreased, leading to the generation of a smaller number of variants, as shown for the BPG treatment group in Table 1. In addition, except for the predominant sequences, many minor variants still existed in each V region, which were concentrated mainly between 1% and 5% (Fig. 2). In general, the diversity of *tprK* in the Amoy strain with multiple sequences was more dispersed after infection, and the diversity of *tprK* variants under normal infection was higher than that in the immunosuppression group. Thus, intervention with penicillin had a significant impact on *tprK* variation.

### Decreasing *tprK* diversity during Amoy strain infection in the three rabbit infection models.

**FIG 3:**
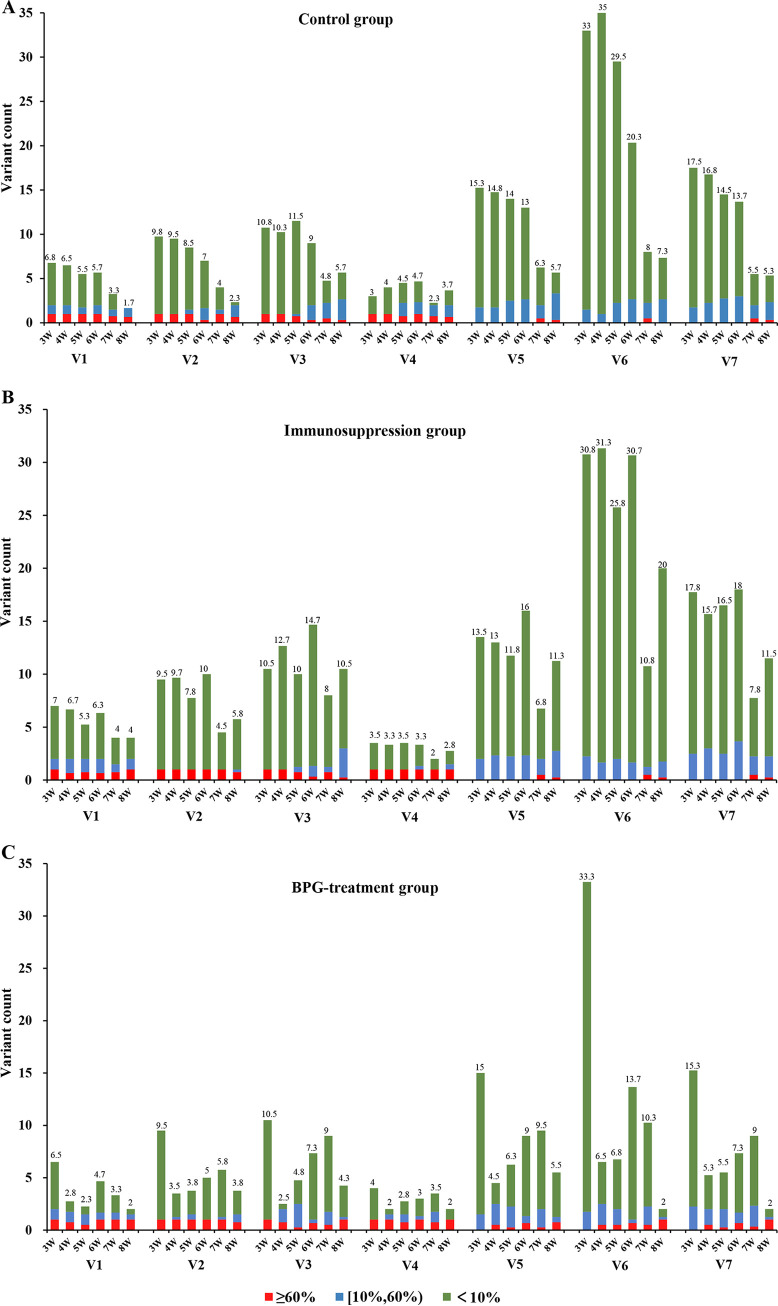
Shown is the decreasing trend of *tprK* gene diversity during Amoy strain infection in three rabbit infection models. The average number of variants with different frequencies in seven V regions of the *tprK* gene over the infection period is shown for the control group (A), the immunosuppression group (B), and the BPG treatment group (C). The red column represents a variant frequency of ≥60%, the blue column represents a variant frequency in the range of (10,60), and the green column represents a variant frequency of <10%. W, week.

Next, we further examined the dynamic variation of *tprK* during Amoy strain infection. At the third week after infection, the number of sequences in each V region was similar in all three groups. The average number of variants of the high diversity V6 was 33.0, 33.3, and 33.8 in the control group, BPG treatment group, and immunosuppression group, respectively (Fig. 3). The number of V region variants then decreased over the infection period, exhibiting a “more to less” trend. In the control group, the typical decreasing trend was observed in the number of distinct V sequences, except in V4 (Fig. 3A). Because of the reduced immune pressure, the average number of variants in the seven V regions showed less frequent variations in the immunosuppression group. Moreover, this group had the highest average number of distinct sequences among the three groups and still exhibited a downward trend of variant number during infection (Fig. 3C). It is noteworthy that this decrease trend was particularly obvious in the BPG treatment group. After the first BPG administration, the number of distinct variants decreased rapidly and then recovered slightly; however, all seven V regions still exhibited a decrease at the eighth week after infection, and the average number of variants was the lowest among the three groups from the fourth week after infection (Fig. 3B). Taking frequency values of ≥60% and ≤10% as the two thresholds between major and minor sequences, we found the proportion of major sequences increased over time in all rabbits, whereas the number and proportion of minor sequences gradually decreased over time (Fig. 3).

**FIG 4:**
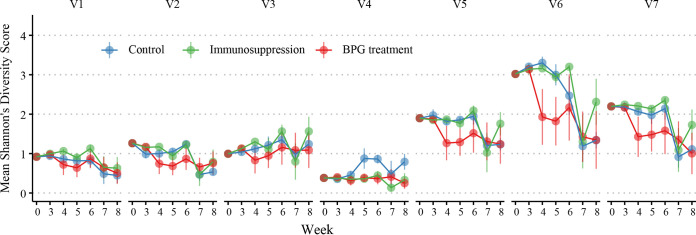
Variability of *tprK* diversity at different time points. Shannon diversity score of samples in different infected rabbits over time. Data are separated by variable regions. Dots represent the mean Shannon diversity score across different color-coded groups, with lines marking the standard deviation. The Wald chi-square test was conducted to identify differences among the three groups at each time point. *P < *0.05 was the significance threshold.

Additionally, we used the Shannon diversity index to analyze the diversity of the *tprK* gene at different time points. The results also revealed a downward trend in *tprK* gene diversity over the infection period, especially in the BPG treatment group. Compared with the other two groups, BPG treatment rabbits exhibited a lower score in this index in six V regions (except for V4) (*P < *0.05) (Fig. 4). Moreover, among the seven V regions, the Shannon diversity index decreased the most for V6 (Fig. 4).

### Persistent existence of predominant V sequences of the *tprK* gene at different infection time points.

**FIG 5:**
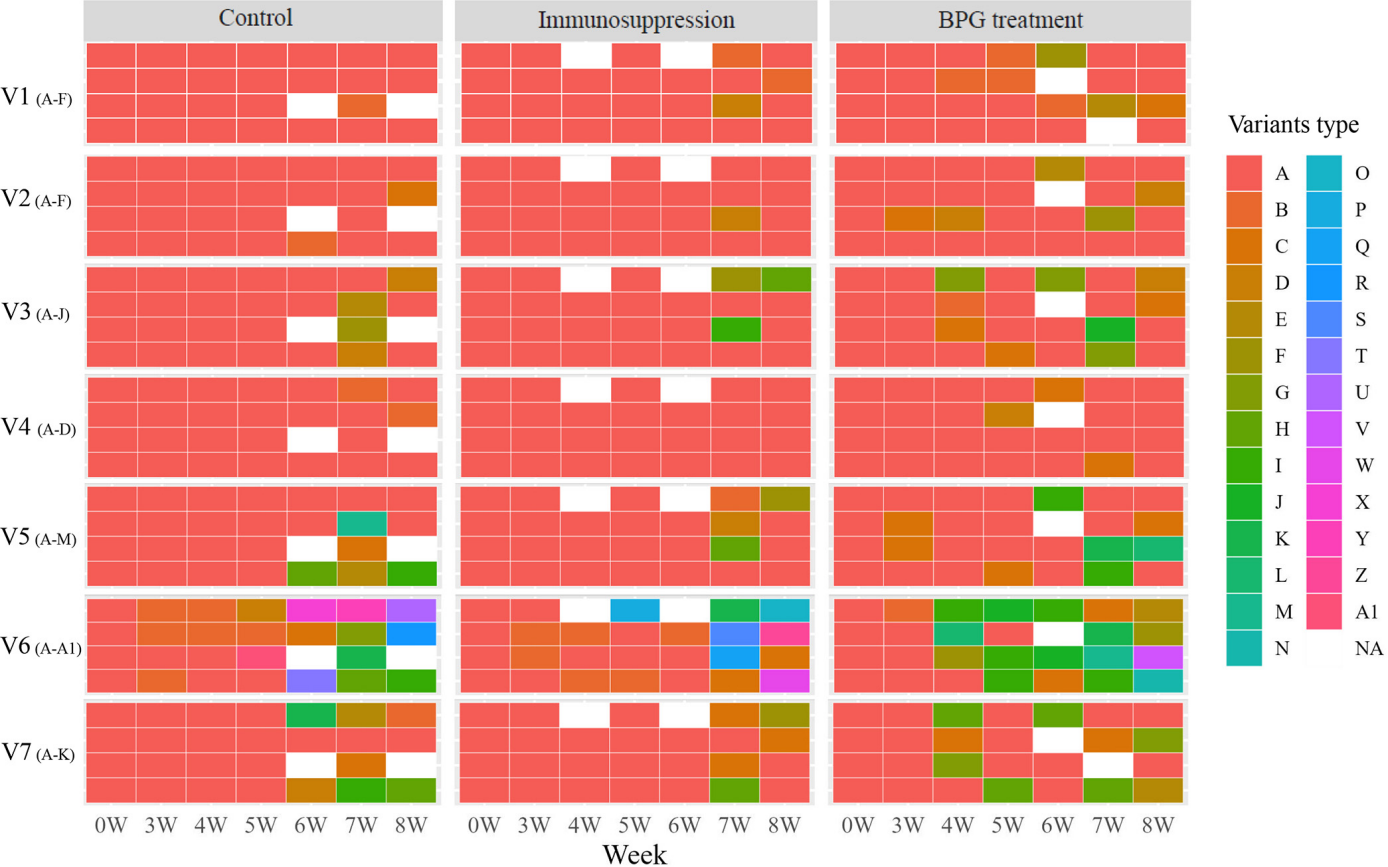
Longitudinal changes of predominant V sequences at different time points within a single rabbit. Only predominant V sequences across infection time points are presented. Variant type A comprises the predominant V sequences of seven regions in the inoculum, whereas variant types from B onward are newly generated predominant V sequences. NA, data not available. The 24 letters (A to A1) represent different predominant variants among 7 V regions, including 6 for V1 (A to F) and V2 (A to F), 7 for V3 (A to J), 4 for V4 (A to D), 13 for V5 (A to M), 27 for V6 (A to A1), and 11 for V7 (A to K).

To better understand the mechanism of V region variation in a single rabbit over time, we longitudinally observed each variant and found that the initial predominant V sequence was prevalent during Amoy strain infection. This phenomenon was observed in the control group (the initial predominant V sequence, marked type A, Fig. 5) and was most obvious in the immunosuppressive group, in which the predominant sequence of six V regions (except for V6) from the third week to sixth week after infection was almost the same as the initial predominant sequence from the inoculum, especially in V1, V2, and V4 regions (Fig. 5). It is worth noting that although the predominant sequence has been persistent, the frequency of this predominant sequence has decreased compared with that of its initial inoculation strain, with many new variants emerging (Table S2). Especially, relatively, most new predominant V sequences occurred in the BPG treatment groups (the new dominant frequency sequences are marked with different colors in Fig. 5). After penicillin administration, the predominant frequency sequence was replaced frequently, particularly in V2, V3, V5, V6, and V7 regions. Nonetheless, the V6 variant was an exception in the seven V regions. Among the 12 rabbits, 27 predominant V6 sequences arose over the infection period. From the third week after infection, the predominant V6 sequence was no longer the initial predominant V sequences. Even in the eighth week, the predominant sequences of V6 in all 12 rabbits changed to new distinct sequences, further proving the diversity of V6 (Fig. 5).

### Immune response between initial predominant V synthesized peptides of TprK and rabbit sera during Amoy strain infection.

Considering that the variation of V regions in the *tprK* gene is related to humoral immunity and that the above results imply the persistent existence of a predominant sequence, we synthesized an amino acid peptide of the initial predominant sequence of the seven V regions in the inoculated strain *in vitro* and detected the immune response against the rabbit sera. As few serum samples corresponding to different time points showed a positive reaction, the enzyme-linked immunosorbent assay (ELISA) results with the highest response among the different time points in a single rabbit were shown in Table 2. The reaction was strongest in the V7 peptide, followed by V4 and V1. In addition, V2 peptides only weakly reacted with the serum of rabbit 11, whereas V5 and V6 did not react at all with the rabbit sera (Table 2). An overall comparison of the ELISA results among the three groups revealed that the control group had the strongest immune response, followed by the immunosuppression group, with the BPG treatment group exhibiting the weakest reaction. Only the sample from rabbit 1 reacted insignificantly to the V1 and V4 peptides (Table 2). We further analyzed the trend of the immune reaction over the duration of infection and found that the reactivity of most peptides initially increased and then decreased over time. The V7 peptide in particular exhibited a positive reaction against sera from the control group after the sixth week and peaked at the eighth week. Then, at the eighth month of euthanasia, the sample/control (S/CO) ratio of ELISA decreased significantly. The reactivity of the V7 peptide also increased initially and then decreased in the immunosuppression group, but the overall S/CO ratio was lower than that in the control group. On the contrary, the V7 peptide almost did not react with the rabbit serum of the BPG treatment group (see Fig. S1 in the supplemental material).

**TABLE 2:** Immune response between initial predominant V synthesized peptides of TprK and rabbits’ serum during Amoy strain infection^*a*^

Peptide	Immune response by group											
	Control group				Immunosuppression group				BPG group			
	No. 1	No. 2	No. 3	No. 4	No. 5	No. 6	No. 7	No. 8	No. 9	No. 10	No. 11	No. 12
V1	−	++	−	+	−	−	−	+	+	−	−	−
V2	−	−	−	−	−	−	+	−	−	−	−	−
V4	−	+	++	−	−	−	+	−	++	−	−	−
V5	−	−	−	−	−	−	−	−	−	−	−	−
V6	−	−	−	−	−	−	−	−	−	−	−	−
V7	++	+	+++	++	−	++	++	+	−	−	−	−

a The synthesis of peptide of V3 region failed, and ELISA detection data was missing. The synthetic peptide was evaluated using ELISA for reaction with rabbits’ sera. Since there were few sera in different timepoints showing positive reaction, only ELISA results with the strongest response among the 13 timepoints in a single rabbit are shown. The optical density (OD) value was measured at 405 nm, and then the ratio of sample/control (S/CO) was calculated. ELISA data were semiquantified as follows: S/CO 0–2.1, – (negative); S/CO 2.1–3, + (weak reactivity); S/CO 3–5, ++ (medium reactivity); and S/CO >5, +++ (strong reactivity).

## DISCUSSION

Heterogeneous *tprK* sequences are hypothesized to be responsible for the persistence of T. pallidum in humans (16, 20). Thus, insights into longitudinal variations in the *tprK* gene throughout the infection process may elucidate the pathophysiology of syphilis. In this study, we analyzed variations in the *tprK* gene in rabbits infected with the Amoy strain, which contains heterogeneous *tprK* sequences, under normal infection and different interventions (immunosuppression and BPG treatment). This study is the first one to employ a wild Amoy strain to establish a syphilis infection model. Consistent with previous reports on patients infected with syphilis (21), an analysis of the variability of the seven V regions in the *tprK* gene revealed that V6 contained the highest number of unique sequences, whereas V4 contained the lowest. However, compared with human samples, the sequences isolated from rabbit lesions infected with the Amoy strain were diverse and more dispersed within a single sample (3, 19). This finding could be related to rabbit passage intensifying the variations in *tprK* (20). Prior research on *tprK* sequence diversity has demonstrated massive upregulation of *tprK* variation after rabbit passage (20). Similarly, in this study, we transmitted the Amoy strain from patients with syphilis to rabbits for conservation and propagation. The change of host inevitably led to an enhancement of variations in the *tprK* gene, resulting in a scattered distribution of distinct sequences.

During 8 weeks of observation, we unexpectedly observed a similar decrease of diversity in *tprK* among all three different rabbit infection models, whereby the frequency of predominant sequences among the seven V regions increased over time (some reaching 100%) and the number of variant sequences progressively decreased, exhibiting a more to less trend. A Shannon diversity analysis further verified this decrease, especially in the BPG treatment group. These findings were contrary to those of previous studies using the Chicago C clone (14, 22, 23), with more variants generated over the duration of the infection. In previous experiments, researchers focused solely on the accumulation of *tprK* diversity in a single subpopulation and ignored the overall trend. However, multiple subpopulations with heterogeneous *tprK* sequences have been observed in the initial clinical infection stage (3), which prompted the use of the Amoy strain containing multiple *tprK* sequences to infect rabbits in this study. As the heterologous Amoy strain with many variants in the seven regions of the *tprK* gene entered the host, survival selection may occur as a result of changes in the host environment, which causes T. pallidum containing the most suitable *tprK* sequence to survive, resulting in a decrease in *tprK* diversity. For the immunosuppression group, the frequency distribution of different variants changed slightly over the six betamethasone injections. The frequency distribution of the *tprK* gene was almost the same as that of the inoculum, which indicated that the host environment at this moment was suitable for the growth of T. pallidum, and there was no need for too many variations. However, after the last betamethasone injection, the frequency of different variants changed greatly. It indicated that after betamethasone withdrawal, the host immune recovered gradually which may cause an increase in survival selection pressure and lead to T. pallidum selecting the most suitable *tprK* sequence to adapt to the current environment, with the rest eliminated, resulting in a decline in diversity as the other two groups at the later infection stage. When T. pallidum contains the most suitable *tprK* sequences, it exhibits relative homeostasis with the host’s immune response; thus, instead of requiring too many variations, these selected *tprK* sequences maintain the basal rate of variation with frequencies of only 1% to 5%, as shown by all three groups exhibiting a more to less phenomenon of *tprK* diversity at the late infection stage. However, this more to less trend was only temporary because the host resistance constantly varied. At 8 months postinfection, we found that the number and diversity of V regions in the *tprK* gene from rabbit testes and lymph nodes increased again (see Table S4 in the supplemental material) and seemed to enter the next round of selection; variations are made for survival, but only a small portion of the *tprK* variant that can adapt to the current environment can survive. This result was consistent with previous research indicating that the tprK gene plays a crucial role in human immune evasion and pathogen persistence (19, 21).

Although the overall tendency of *tprK* diversity was different from that in previous studies (14, 22, 23), we found that immune pressure still plays an important role in *tprK* variation during Amoy strain infection, as reflected by the lower variant number of the *tprK* gene in the immunosuppression group. Previous studies have shown that during infection, TprK is targeted by the host immune response, with T cells responding to the conserved regions, while the humoral immune response targets the V regions (18). Weakening of the humoral immune response can slow down mutation in the variable regions of TprK (22) as presented in our immunosuppressed group. Additionally, we analyzed the *tprK* diversity after BPG treatment and found that the BPG treatment group possessed the smallest number of variants, as well as the lowest Shannon index among the three groups. This finding can be attributed to the fact that T. pallidum in patients with syphilis undergoing the recommended therapy is eliminated by penicillin-mediated killing, and the role of the host immune clearance is relatively weak, so the immune pressure against *tprK* variation is also weak (24). Furthermore, our results showed that the predominant sequence with the highest frequency was replaced frequently in V2, V3, V5, V6, and V7 in the BPG treatment group, suggesting that many different variants contribute to resistance against the host’s clearance. This diversity variants might increase the probability of pathogen survival. If the pathogen is not cleared completely at this time, the pathogen could easily escape the immune response and revive. Thus, compliance with the standard syphilis treatment is very important (25). It is worth noting that although the *tprK* sequence was able to be detected after BPG treatment, the rabbit infection tests (RIT) conducted 8 months after infection revealed that 0% (0/4) of the BPG treatment group showed positive RIT, while 75% (3/4) of the immunosuppression group and 66.67% (2/3; 1 rabbit died unexpectedly during the experiment) of the control group showed positive RIT. The results could demonstrate that BPG treatment was complete.

By analyzing the predominant sequence in a single sample, we found that, although *tprK* varied during the infection period, the original predominant V region sequence did not disappear completely and remained prevalent. Therefore, we further synthesized the protein peptides encoded by the predominant sequence in each V region and analyzed its immune response using ELISA. The results showed that V7 presented the strongest immune response, whereas V4 and V1 presented weak reactions. The reaction of the V7 peptide against the rabbit serum antibody increased over time but decreased in the eighth month after infection, which indirectly suggests that variability in *tprK* may permit evasion of the antibody response of the infected host. Contrary to previous studies that showed an antibody response in V2, V4, V5, V6, and V7 in rabbit models (14), we observed positive reactions in V1, V4, and V7. The main reason for this difference is that certain V region base sequences may be part of a conformational epitope and anti-V antibodies fail to recognize and bind the short linear peptides detected through ELISA (14, 26). Furthermore, the predominant sequence that persists during infection may indicate long-term evolutionary selection, which could help T. pallidum resist the immune system. Thus, cocktail TprK-related vaccines can be designed with different predominant frequency sequences, instead of being limited to use of the Nichols reference strain. It needs to be pointed out that although the predominant sequence had been consistently present, its frequency had decreased and new mutant variants emerged, which can help immune escape and lead to long-term infection.

Admittedly, our study has some shortcomings. First, we analyzed the samples only during the first 8 weeks; therefore, variations in *tprK* that may have been produced after 8 weeks are unknown. Secondarily, although we have selected high and low *T. pallidum* load samples from different time points in the BPG treatment group for *tprK* gene analysis and the diversity of the variable region in the *tprK* gene was highly consistent between the two independent assays indicating the influence of *T. pallidum*-DNA load on the diversity of *tprK* was not critical, there was still lacking direct data to support it. Further experiments are needed to obtain more data to eliminate this doubt. Third, all skin lesions were sampled *in situ*; however, the results would be more representative if we were able to analyze *tprK* in the distal organs. Moreover, we analyzed the variations of *tprK* based on the DNA level instead of based on the RNA level which may not reflect the more instant variations of the *tprK* gene. Additionally, we provided information only on individual V regions; thus, the study lacks data on the profile of full-length *tprK*.

In general, the findings of this study indicate that survival selection may be another internal factor that promotes the variation of *tprK*, leading to a reduction in *tprK* diversity during Amoy strain infection. BPG treatment could reduce the diversity of *tprK* but also leads to more frequent changes of the predominant sequence, which could increase the probability of pathogen survival. The persisting predominant sequence of each V region may present a new direction for TprK-related vaccine research.

## MATERIALS AND METHODS

### Ethics statement.

All animal experiments adhered to the procedures outlined in the Guide for the Care and Use of Laboratory Animals approved by the Zhongshan Hospital Institutional Animal Care and Use Committee.

### Treponema pallidum Amoy strain.

The T. pallidum Amoy strain is a SS14-like wild strain isolated from patients with primary syphilis in our laboratory, with complete genetic information available (9). This strain exhibits minimal passage in rabbits and retains the natural aspects of the pathogenesis of syphilis, such as persistent infection, antigen variation, and immune escape (27). In contrast to Nichols and Chicago C strains, which are near clones used commonly in previous *tprK* studies (14), the Amoy strain has multiple *tprK* gene sequences, which indicates a high degree of heterogeneity in this strain. This characteristic is closer to the isolated clinical strains.

### Construction of rabbit models of normal infection and immunosuppression and BPG intervention using the Amoy strain.

New Zealand rabbits were inoculated intradermally on the back with the Amoy strain. First, we inoculated the rabbit testis with Amoy strain frozen in 2013 and then extracted spirochetes after three generations. Twelve male New Zealand rabbits were infected intradermally with 10^5^ Amoy treponemes per site on their clipped back at 10 different sites. Then, rabbits were divided randomly into three groups of four, which were designated the normal infection (control group), immunosuppression treatment, and BPG treatment groups. The control group did not receive any additional treatment, whereas the immunosuppression group was injected intramuscularly with 20 mg of compound betamethasone in six doses administered weekly (18, 22). To construct the BPG treatment group, once the lesion reached its maximum diameter in the third week after infection, 200,000 units of BPG (Zhongnuo Pharmaceutical Co., Ltd., Shijiazhuang, China) were injected intramuscularly into the inner thigh muscle of the rabbits once per week for a period of 2 weeks (27) (Fig. 1). All rabbits were monitored for skin lesion development every day with blood drawn at 0, 3, 7, 9, 11, 13, 17, and 21 days (3 weeks) and later weekly until the skin lesions subsided. Serum samples were stored at −80°C for subsequent enzyme linked immunosorbent assay (ELISA) detection of the V region-specific antibody response. Cutaneous lesion biopsy specimens were harvested from each rabbit using a 4-mm biopsy punch for DNA extraction and *tprK* sequencing from the third to eighth week after infection. Finally, 8 months postinfection, blood was drawn from 11 rabbits and analyzed using ELISA (one rabbit died in control group during the experiment). All rabbits were then euthanized. The popliteal lymph nodes and testicular tissues were harvested aseptically, extracted in saline, and inoculated into naive anesthetized rabbit testes as described previously (27, 28) (Fig. 1).

### Quantitative PCR and NGS.

The DNA from skin lesions, lymph nodes, and testicular tissues was extracted and tested for *T. pallidum* load using the *Tp47* gene as described previously (29). NGS and data extraction of the *tprK* gene were conducted according to our previously described method (3). Briefly, library construction and sequencing were performed by the Sangon Biotech Company (Shanghai, China) on the MiSeq-PE300 instrument (Illumina, San Diego, CA) in paired-end bidirectional sequencing mode. The quality of the raw data was checked and improved by the FastQC and FasTX tools. The Bowtie 2 (version 2.1.0) was used to compare the reads with the tprK gene of the Seattle Nichols strain (GenBank number AF194369.1). Based on the previously published principle that was used to extract variable regions (10), an in-house Perl script was developed to specifically capture the sequences in the seven variable regions of the tprK gene. In brief, the defined strings (approximately 12 to 16 bp) that matched the conserved sequence of the tprK gene were used to capture different V region sequences. Because of the minor mutations in the 5′-flank region of V3 in the Amoy strain, two newly defined sequence strings were designed for the V3 region (see Table S1 in the supplemental material). All variant sequences within each variable region that met the following two criteria were included in this study: (i) had at least 50 reads and (ii) had a frequency above 1%. Finally, the relative frequency of the variant sequences within each variable region in a single sample was calculated as in Table S2.

### Immunoassays using synthetic peptides of TprK encoded by predominant sequence variants in the inoculum of the Amoy strain.

The V region-specific antibody response was tested through ELISA. First, predominant sequence variant peptides of TprK in the inoculum of the Amoy strain were synthetized by the Sangon Biotech Company (Shanghai, China) (see Table S3 in the supplemental material). Then, each peptide was diluted in carbonate buffer (0.1 mol/L NaHCO_3_ [pH 9.6]) to a concentration of 10 μg/mL, and 100 μL of peptide buffer was incubated in 96-well plates at 4°C overnight. The plates were then washed and blocked as described in a previous study (30). Infected rabbit sera isolated at different time points were diluted in phosphate-buffered saline to a final concentration of 1:80. The blank hole that was not coated with peptide was defined as the negative control. Absorbance was measured at 405 nm, and the mean ± standard error of triplicate wells was calculated for every V region peptide against the sera from all infected rabbits at different time points.

### Data analysis.

Statistical analysis was performed using SPSS software (SPSS Inc., Chicago, IL). The Shannon diversity index, describing community diversity, was used in this study to evaluate the diversity of seven V regions of *tprK* in a single sample, and its diversity scores were calculated by using the R package VEGAN (21) and were presented as the mean ± SD. The Wald chi-square test was conducted to identify differences among the three groups at each time point. A *P* value of <0.05 was considered statistically significant. Data visualization analysis was performed using R language.

### Data availability.

The raw data obtained from the *tprK* sequencing in this study are available under NCBI BioProject number PRJNA943511.
