# Supplementary material for: Longitudinal Variations in the tprK Gene of Treponema pallidum in an Amoy Strain-Infected Rabbit Model
Source: Microbiol Spectr. 2023 Jun 22;11(4):e01067-23. doi: 10.1128/spectrum.01067-23 (PMC10433980; doi:10.1128/spectrum.01067-23)
Supplement: Supplemental file 1 — Supplemental material. Download spectrum.01067-23-s0001.docx, DOCX file, 0.01 MB [file spectrum.01067-23-s0001.docx]

**Supplementary data**

**Figures**

**Figure S1. Dynamic changes in the antibody reaction between V7 peptide and serum over different timepoints**

Three experimental groups were used in this study: control group, BPG treatment group, immunosuppression group. BPG: benzathine penicillin G, d: day, w: week, M: month.

**Tables**

**Table S1. Defined strings used to capture the seven V regions**

**Table S2. Detailed information of different variant sequences within the seven variable regions (V1–V7) of the *tprK* gene captured directly from 68 samples enrolled in this study**

control group (1, 2, 3, 4), immunosuppression group (5, 6, 7, 8), BPG treatment group (9, 10, 11, 12). "(N1)W-(N2)": N1 represents the week and N2 represents the rabbit number; therefore, (N1)W-(N2) represents the skin lesions from rabbit N2 at week N1 and 8W-12 represents the skin lesions from rabbit 12 at week 8. "-F" refers to the relative frequency of different variants. "-": data not available. "Inoculum": extracted spirochetes after resuscitation used for the back intradermal inoculation of 12 rabbits.

**Table S3. Amino acid sequences of the initial predominant sequence in different V regions**

**Table S4. Detailed information of seven variable regions (V1-V7) of the *tprK* gene can be detected in the lymph nodes and testicular tissues of rabbits after eight months of infection.**

"-F" refers to the relative frequency of different variants.

The *tprK* sequencing was performed on the lymph nodes and testicular tissues of all 11 rabbits (one rabbit died in control group during the experiment) after eight months of infection, but only samples from five rabbits were able to get the tprK sequence information
