# Supplementary figures and images for: Longitudinal Variations in the tprK Gene of Treponema pallidum in an Amoy Strain-Infected Rabbit Model
Source: Microbiol Spectr. 2023 Jun 22;11(4):e01067-23. doi: 10.1128/spectrum.01067-23 (PMC10433980; doi:10.1128/spectrum.01067-23)

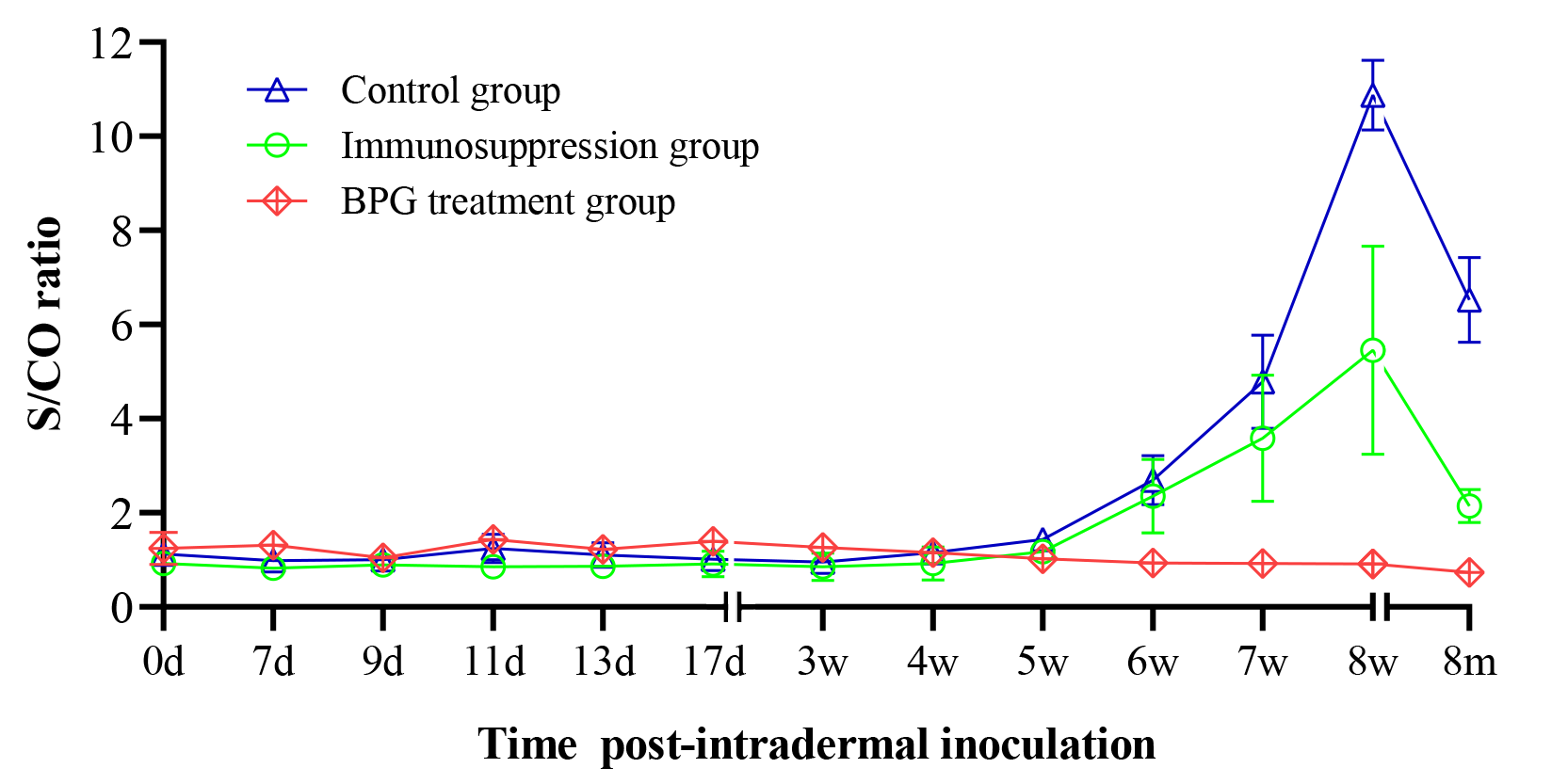

Supplement: Supplemental file 2 — Supplemental material. Download spectrum.01067-23-s0002.tif, TIF file, 0.3 MB [file spectrum.01067-23-s0002.tif]
